# Fulminant Type 1 Diabetes in Children: A Multicenter Study in China

**DOI:** 10.1155/2017/6924637

**Published:** 2017-09-26

**Authors:** Yi Gu, Yi Wang, Pin Li, Haiyan Wei, Linqi Chen, Qianqi Liu, Yu Liu, Qiaozhi Yang, Xinran Cheng, Lanjie He, Liya Wei, Zhiying Zhu, Yongxing Chen, Fengyun Wang, Xing Shi, Yuxian Cheng, Yan Wei, Jianing Yu, Chunxiu Gong

**Affiliations:** ^1^National Key Discipline of Pediatrics, Department of Endocrinology, Genetics and Metabolism, Beijing Children's Hospital, Capital Medical University, National Center for Children's Health, Beijing 100045, China; ^2^Department of Microbiology and Immunology, Dartmouth College, Dartmouth-Hitchcock Medical Center, Lebanon, NH 03756, USA; ^3^Department of Endocrinology, Children's Hospital of Shanghai, Shanghai Jiao Tong University, Shanghai 200062, China; ^4^Department of Endocrinology and Genetic Metabolism, Zhengzhou Children's Hospital, Zhengzhou 450053, China; ^5^Department of Endocrinology, Children's Hospital of Soochow University, Suzhou 215003, China; ^6^Department of Endocrinology, Nanjing Children's Hospital of Nanjing Medical University, Nanjing 210005, China; ^7^Department of Endocrinology, Genetics and Metabolism, The Children's Hospital of Guiyang City, Guiyang 550003, China; ^8^Department of Pediatric, Liaocheng Children's Hospital, Shandong 252002, China; ^9^The Pediatric Endocrine Department, Chengdu Woman and Children's Center Hospital, Chengdu 610091, China; ^10^Department of Pediatric, General Hospital of Ningxia Medical University, Yinchuan 750004, China

## Abstract

**Background:**

To investigate the hospital-based incidence of FT1D in Chinese children and compare the clinical feature with classical T1DM.

**Methods:**

A cross-sectional study with sixteen hospitals involved. We obtained 23 FT1D cases as group 1, acute-onset T1DM as group 2, and typical T1DM as group 3.

**Results:**

The incidence of FT1D was 1.56% in 16 participating hospitals. The mean age at the onset of group 1 was 2.00 (1.08, 6.51) years old, much younger than that of group 2 (6.11 (3.92, 9.50)) and group 3 (6.92 (4.17, 10.03)). In addition, significant differences were found between three groups: mean BMI and flu-like symptoms with fever and abdominal pain. Follow-up comparison of three groups from Beijing Children's Hospital for at least one year showed that there is no significant difference between the three groups in terms of mean HbA1c levels and insulin injection dosages.

**Conclusion:**

FT1D onset age is much younger than that of classical T1D patients. The hospital-based incidence of FT1D in Chinese children was 1.56% in all new-onset T1DM. For the diagnosis, making FT1D alone into a subtype within type 1 diabetes may be meaningful. However, for the treatment and prognosis, such classification should not be helpful to the clinic.

## 1. Background

Fulminant type 1 diabetes (FT1D) was first reported by Imagawa et al. in 2000 [1]. Most reported cases are from East Asia and occur during pregnancy or just after delivery [1, 2]. This type of diabetes is defined as a subtype of type 1 diabetes (T1DM) because it is considered as having a different mechanism from classical T1DM [1, 3, 4]. Since the recognition of FT1D, It had been reported in Korean [5–8], Chinese [9], French [10], and US Hispanic patients [11]. However, there are no data on children (below 15 years old). To clarify the more detailed clinical characteristics and difference between FT1D and classical T1DM in children in China, we performed a multicenter study with 16 hospitals.

## 2. Methods

### 2.1. Patients

**Figure 1:**
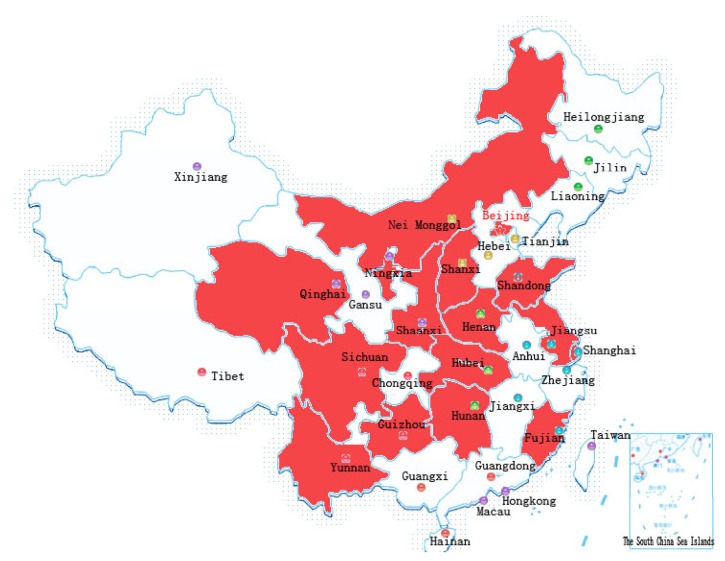
The location of each hospital of this study in China.

It is a cross-sectional study with a multicenter design including sixteen hospitals. But in terms of Beijing Children's Hospital, it should be a cohort study. All patients were diagnosed within January 2004 to December 2012. One dedicated doctor was in charge of reviewing all the data and 2 other doctors in auditing according to the same criterion. Finally, we got effective data from nine hospitals. Seven cases (30.43%) and 16 (69.57%) cases were from south and north, respectively (see Figure 1).

Groupings: group 1 included 23 fulminant type 1 diabetes cases. The clinical characteristics of FT1D were [10] (1) remarkably abrupt onset; (2) very short (<1 week) duration of diabetic symptoms (thirst, weight loss, and polyuria); (3) occurrence of diabetic ketosis or ketoacidosis soon (approximately 7 days) after the onset of hyperglycemic symptoms (elevation of urinary and/or serum ketone bodies at first visit); (4) plasma glucose level ≧ 16.0 mmol/L (‡288 mg/dL) and HbA1c < 8.5% (Japan Diabetes Society value) at first visit; and (5) urinary C-peptide excretion < 10 *μ*g/d or fasting serum C-peptide level < 0.3 ng/mL (<0.10 nmol/L) and <0.5 ng/mL (<0.17 nmol/L) after intravenous glucagon (or after meal) load at onset. Other findings in FT1D were (1) flu-like symptoms (fever, upper respiratory symptoms, etc.) or gastrointestinal symptoms (upper abdominal pain, nausea and/or vomiting, etc.). The second group consisted of 182 acute-onset type 1 diabetes cases where (1) patients meet the criteria of the International Diabetes Federation (IDF) and International Society of Pediatric and Adolescent Diabetes (ISPAD) for type 1 diabetes; (2) there is presence of ketosis or ketoacidosis at the onset of diabetes; and (3) the onset of diabetic symptoms was less than 30 days. Group 3 consists of 879 typical type 1 diabetes cases who had diabetic symptoms within 30–100 days. The study program was approved by the ethical committee of the Beijing Children's Hospital.

### 2.2. Index of Clinical Characteristics and Biochemical Analysis

Data at admission clinically included onset age, sex, hyperglycemic symptom duration, family history of diabetes in first-degree relatives, influenza-like symptoms, blood pressure, and body mass index. Laboratory tests included blood glucose, glycosylated hemoglobin (HbA1c), arterial pH, bicarbonate, *β*-hydroxybutyric acid, electrolytes, aspartate aminotransferase, alanine aminotransferase, total cholesterol, and triglyceride. Glutamic acid decarboxylase antibodies (GADAb), insulin autoantibodies (IAA), and islet cell antibodies (ICA) in serum samples were determined with the enzyme-linked immunosorbent assay method. And fasting plasma C-peptide and 2-h postprandial C-peptide levels were determined using the electrochemiluminescence immunoassay method after the resolution of diabetic ketoacidosis.

Some data were missing in other 7 hospitals; we followed up a comparison of three groups of Beijing Children's Hospital.

### 2.3. Statistical Analysis

Use SPSS17.0 software. Analysis of variance or Kruskal–Wallis H test was used. Group comparisons were done by using least significant difference test. Frequency comparisons were done by using Fisher's exact test. All continuous variables with a normal distribution are expressed as means ± standard deviation. All tests were two-sided, and a *P* < 0.05 was required for statistical significance.

## 3. Results

### 3.1. General Information

There were 23 patients diagnosed with FT1D since 2004–2012, 9 males and 14 females. The incidence of FT1D was 1.56%. Mean age at the onset of group 1 was 2.00 (1.08, 6.51) years old, which was much younger than that of group 2 at 6.11 (3.92, 9.50) years old and group 3 at 6.92 (4.17, 10.03) years old. Significant differences were found between three groups in the mean BMI 16.12 (14.51, 19.55) versus 15.01 (13.54, 17.29) and 14.87 (13.61, 16.64). Abdominal pain was observed in twelve patients (52.2%), much more than that in group 2 (17.6%) and group 3 (7.5%) (see Table 1).

### 3.2. Biochemical Analysis

Mean plasma glucose in group 1 was higher (25.10 (20.35, 30.00)) than that in group 2 (22.99 (17.99, 30.89)) and group 3 (21.78 (15.70, 28.96)). Similar results were found in triglycerides: 1.24 (0.86, 1.59) versus 1.45 (0.90, 2.52) and 1.10 (0.75, 1.83). There was also significantly lower arterial blood pH and lower plasma bicarbonate concentrations (*P* = 0.001) (see Table 1).

For incidence of acute complications of the three groups, a significant difference was found in group 1 in low T3 syndrome, higher than the other two groups (*P* = 0.018). Other acute complications such as serious DKA, HHS, rhabdomyolysis, myocardial damage, and encephaledema were not observed as different between the three groups (see Table 2).

### 3.3. Follow-Up Results

Follow-up comparison of three groups from Beijing Children's Hospital showed that there is no significant difference in these three groups neither in mean HbA1c levels nor in insulin injection dosages. Particularly, three groups of patients used minimal insulin injection dosages to maintain plasma glucose level in honeymoon period (see Table 3).

## 4. Discussion

The prevalence of FT1D worldwide is different. 19.4% of acute-onset type 1 diabetes was revealed in Japan [12] and 7.1% of newly diagnosed type 1 diabetic patients in Korea [6]. Luo et al. [13] reported 53 cases of FT1D from 24 hospitals nationwide in China, and the percentage of FT1D was 14.9%. However, the patients in the above research were older children and adults. The minimum age of patients enrolled in those researches was 12 years old. The mean age at onset was 35 years in females and 43 years in males; 91.3% of patients were adults and pregnancy is associated with female fulminant type 1 diabetes [10]. In this study, we collected 23 FT1D patients younger than 15 years who were children from different provinces of China. The incidence of FT1D was 1.56% among all newly T1DM patients, much lower than in adults reported, but it is similar to those Korean studies which reported that the frequency of FT1D was 1.33% under the age of 16 years [8]. The mean age at onset was 2 years (1.08, 6.51). It is quite younger than that in adults, suggesting that the disease occurs in younger age groups of children.

FT1D patients displayed a more diverse clinical manifestation, including flu-like symptoms and gastrointestinal discomforts. In our study, flu-like symptoms and fever were observed in seventeen cases (73.9%), much more than acute-onset type 1 diabetes group (10.4%) and typical type 1 diabetes (4.5%). Also, abdominal pain was observed in twelve patients (52.2%), much more than acute-onset type 1 diabetes group (17.6%) and typical type 1 diabetes group (7.5%). These features are similar to those reported in Koreans and Japanese [6, 14]. Specially, FT1D and acute-onset type 1 diabetes are both acute-onset diseases, but FT1D with flu-like symptoms and abdominal pain was much more frequent than acute-onset T1D. This result indicated that the contribution of viral infection is important to predispose or trigger FT1D. Otherwise, this also seems to explain why the disease occurs in younger age groups of children.

Pathogenesis of FT1D is not clear. Early studies indicated no evidence of islet autoimmunity in FT1D, while recent findings have increasingly suggested that islet autoimmunity is involved in its development [15–17]. Autoantibodies against the *β*-cell antigens, such as GAD (two patients), insulin cell autoantibodies (one patient), and islet cell antoantibodies (one patient), were found in these twenty-three FT1D patients. This result accounts for 20% positive and also has a similar literature report [18–20]. It was given that T-cell-mediated autoimmunity played the destruction role of the pancreatic *β*-cells [21, 22]. It is possible that *β*-cell-specific Th1 immunity, together with low-grade humoral immune responses, further disposes patients to the development of FT1D. In addition, genetic factors also play a role in the pathogenesis. The HLA class II genes, especially HLA-DQ and HLA-DR, have been associated with a high susceptibility of individuals to autoimmune type 1 diabetes [22]. The DR4-DQ4 genes are also associated with the development of FT1D in Japan, and higher frequencies of HLA-DRB1^∗^0405-DQB1^∗^0401 or HLA-DQA1^∗^0303-DQB1^∗^0401 and HLA-DQA1^∗^0302-DQB1^∗^0303 haplotypes are observed in Japanese fulminant patients [23–27].

Recently, hypothesis of FT1D is thought to be a coefficient of viral infection and genetic factors [28]. Viral infections will accelerate antiviral immune reactions of CTLA-4, which induce *β*-cell death [3]. Moreover, environmental insults such as viral infections are related to alter immune responses in periphery and around the islet [29]. Cytokines such as interleukin and tumor necrosis factor-*α* recruit additional T cells, macrophages, and NK cells to the islets, and their signaling transductions have direct cytotoxic effects on *β*-cells. The discovery of infiltrating around and in the islet indicates that FT1D may experience a similar pathogenic process as classic type 1 diabetes [30]. In addition to this, Aida et al. [31] studied the in situ status of innate and adaptive immunity of enterovirus-induced FT1D. RIG-I was strongly expressed in *β*-cells in pancreas infected with enterovirus. T lymphocyte receptors (TLR3) were expressed in mononuclear cells that infiltrated islets. Interferon-*α* (IFN-*α*) and IFN-*β* were strongly expressed in islet cells. Major histocompatibility complex (MHC) class I, IFN-*γ*, interleukin-18, and Cytotoxic C motif ligand 10 were expressed and colocalized in affected islets. Serum levels of IFN-*γ* were markedly increased in patients with T lymphocyte receptor [32]. These findings demonstrate the presence of specific innate immune responses to enterovirus infection in T lymphocyte receptor. Therefore, the diagnosis of idiopathic diabetes in FT1D is still to be determined. More discussion and accumulation of cases are essential to conclude whether autoimmunity is involved in FT1D.

FT1D patients had significantly lower arterial blood pH and lower plasma bicarbonate concentrations. Meantime, the serum triglyceride was higher than that in typical type 1 diabetes but not seen in other groups. Otherwise, there is no obvious difference of three groups in acute complications. All these results indicate that FT1D patients display a more diverse clinical manifestation. Significant higher mean BMI was found in FT1D. This related higher BMI phenomenon may be associated with short duration of body weight loss.

There is no significance different in these three groups from Beijing Children's Hospital neither in mean HbA1c levels nor in insulin injection dosages when followed up. Particularly, there is almost the same minimal insulin dosage per day to maintain plasma glucose level in honeymoon period. Therefore, FT1D are similar as acute-onset diabetes and typical type 1 diabetes in insulin treatment and prognosis in children. Indeed, there are some special cases reported which presented another profile, such as Yamashita et al. [33] who reported a woman after acute pancreatitis and FT1D developed simultaneously. She experienced transient complete remission of diabetes and eventually had mild diabetes with non-insulin-dependency and impaired insulin secretion.

In conclusion, this study showed that the incidence of FT1D below 15 years old was very low, the incidence was 1.56%, and the age of FT1D onset in childhood is much younger. Considering the incidence of this disease and no significant difference of FT1D and type 1 diabetes (mean HbA1c levels, injection dosages, and minimal insulin injection dosages to maintain plasma glucose level in honeymoon period), we elicit that for the diagnosis, making FT1D alone into a subtype within type 1 diabetes may be meaningful. However, for the treatment and prognosis, such classification should not be helpful to the clinic, while for the limitation of a small size in following up, more follow-up work should be done.

## Figures and Tables

**Table 1:** Clinical features and laboratory tests of the three groups.

	F1TD (9/14)	Acute onset T1DM (83/99)	Classical T1DM (375/504)	*F*	*P*
Duration of hyperglycemia (days)	7 (2, 10)	7 (5, 8)	15 (10, 25)	234.736	0.000^#^
Age (year)	2.00 (1.08, 6.51)	6.11 (3.92, 9.50)	6.92 (4.17, 10.03)	14.048	0.001^#^
BMI (kg/m^2^)	16.12 (14.51, 19.55)	15.01 (13.54, 17.29)	14.87 (13.61, 16.64)	4.621	0.099
Family history	0	12	32	—	—
Flu-like symptoms	17	19	40	57.702	0.000^#^
Abdominal pain	12	32	66	29.12	0.000^#^
Plasma glucose (mmol/L)	25.10 (20.35, 30.00)	22.99 (17.99, 30.89)	21.78 (15.70, 28.96)	9.765	0.008^#^
HbA1C (%)	7.2 ± 1.2	11.7 ± 2.1	11.8 ± 2.4	43.909	0.000^#^
Peptide C (ng/mL)	0.21 (0.09, 0.29)	0.31 (0.11, 0.54)	0.34 (0.13, 0.58)	8.464	0.015^#^
Diastolic blood pressure (mmHg)	60 (60, 75)	60 (60, 70)	60 (60, 70)	0.619	0.734
Systolic pressure (mmHg)	90 (70, 103)	100 (90, 110)	100 (90, 110)	5.026	0.081
pH	7.23 (6.98, 7.34)	7.28 (7.14, 7.38)	7.32 (7.19, 7.40)	14.346	0.001^#^
HCO_3_ (mmol/L)	8.20 (3.10, 16.85)	13.10 (7.85, 18.90)	16.30 (8.90, 22.10)	16.657	0.000^#^
*k* (mmol/L)	4.41 (4.00, 4.91)	4.10 (3.74, 4.60)	4.12 (3.74, 4.51)	3.792	0.150
Na (mmol/L)	135.6 (131.0, 138.0)	135.0 (131.1, 136.9)	135.0 (131.6, 138.0)	2.602	0.272
Cl (mmol/L)	105.0 (98.0, 107.0)	103.2 (100.0, 107.5)	103.2 (99.2, 106.8)	0.27	0.874
Plasma osmolal pressure (Mosm/L)	284.8 (276.7, 293.1)	288.8 (281.3, 297.0)	289.4 (282.8, 296.6)	2.817	0.244
BUN (mmol/L)	4.20 (2.70, 5.84)	4.48 (3.39, 6.17)	4.60 (3.50, 5.88)	1.437	0.488
CHO (mmol/L)	3.59 (3.24, 5.05)	3.99 (3.32, 4.98)	4.12 (3.44, 4.90)	1.015	0.602
TG (mmol/L)	1.24 (0.86, 1.59)	1.45 (0.90, 2.52)	1.10 (0.75, 1.83)	14.545	0.001^#^
AST (U/L)	30 (22, 33)	21 (15, 27)	22 (18, 29)	8.159	0.017^#^
ALT (U/L)	16 (12, 23)	15 (12, 18)	15 (12, 20)	0.808	0.668
Insulin dosage of ketoacidosis treatment (IU)	11.93 (7.42, 31.35)	13.20 (8.40, 25.89)	14.43 (9.26, 28.00)	1.333	0.513
Time of ketoacidosis treatment (hour)	15.0 (4.5, 21.5)	9.3 (6.0, 15.6)	10.0 (6.0, 16.5)	1.043	0.594

The statistical method used was analysis of variance. # represents *P* < 0.05.

**Table 2:** Acute complications of three groups.

	Serious DKA	HHS	Low T3 syndrome	Rhabdomyolysis	Myocardial damage	Encephaledema
Group 1	4	0	4	0	1	0
Group 2	19	1	10	0	3	0
Group 3	76	2	89	0	30	0
*x* ^2^	5.065	0	8.06	—	1.986	—
*P*	0.079	—	0.018^#^	—	0.371	—

^#^There exist differences between group 1 and group 2 and between group 1 and group 3.

**Table 3:** Follow-up comparison of three groups from Beijing Children's Hospital.

	Course of disease: one month		Course of disease: six months		Course of disease: one year	
	HbA1C (%)	Insulin dosage (IU/kg/d)	HbA1C (%)	Insulin dosage (IU/kg/d)	HbA1C (%)	Insulin dosage (IU/kg/d)
Group 1 (*n* = 5)	7.9 ± 1.2	0.61 ± 0.19	7.1 ± 1.0	0.58 ± 0.12	7.5 ± 1.1	0.63 ± 0.13
Group 2 (*n* = 25)	8.2 ± 1.2	0.51 ± 0.32	7.8 ± 2.1	0.58 ± 0.31	7.5 ± 1.6	0.49 ± 0.31
Group 3 (*n* = 65)	8.3 ± 1.4	0.59 ± 0.35	7.3 ± 1.6	0.58 ± 0.33	7.6 ± 1.7	0.58 ± 0.35
*x* ^2^	0.170	0.555	0.889	0.000	0.013	0.726
*P*	0.844	0.576	0.415	1.000	0.987	0.487
